# RAGE Regulates Immune Cell Infiltration and Angiogenesis in Choroidal Neovascularization

**DOI:** 10.1371/journal.pone.0089548

**Published:** 2014-02-26

**Authors:** Mei Chen, Josephine V. Glenn, Shilpa Dasari, Carmel McVicar, Michael Ward, Liza Colhoun, Michael Quinn, Angelika Bierhaus, Heping Xu, Alan W. Stitt

**Affiliations:** 1 Centre for Experimental Medicine, Queen’s University of Belfast, Belfast, United Kingdom; 2 Department of Medicine and Clinical Chemistry, University of Heidelberg, Heidelberg, Germany; Institut de la Vision, France

## Abstract

**Purpose:**

RAGE regulates pro-inflammatory responses in diverse cells and tissues. This study has investigated if RAGE plays a role in immune cell mobilization and choroidal neovascular pathology that is associated with the neovascular form of age-related macular degeneration (nvAMD).

**Methods:**

RAGE null (RAGE−/−) mice and age-matched wild type (WT) control mice underwent laser photocoagulation to generate choroidal neovascularization (CNV) lesions which were then analyzed for morphology, S100B immunoreactivity and inflammatory cell infiltration. The chemotactic ability of bone marrow derived macrophages (BMDMs) towards S100B was investigated.

**Results:**

RAGE expression was significantly increased in the retina during CNV of WT mice (p<0.001). RAGE−/− mice exhibited significantly reduced CNV lesion size when compared to WT controls (p<0.05). S100B mRNA was upregulated in the lasered WT retina but not RAGE−/− retina and S100B immunoreactivity was present within CNV lesions although levels were less when RAGE−/− mice were compared to WT controls. Activated microglia in lesions were considerably less abundant in RAGE−/− mice when compared to WT counterparts (p<0.001). A dose dependent chemotactic migration was observed in BMDMs from WT mice (p<0.05–0.01) but this was not apparent in cells isolated from RAGE−/− mice.

**Conclusions:**

RAGE-S100B interactions appear to play an important role in CNV lesion formation by regulating pro-inflammatory and angiogenic responses. This study highlights the role of RAGE in inflammation-mediated outer retinal pathology.

## Introduction

The early stages of age-related macular degeneration (AMD) are characterised by progressive dysfunction of the retinal pigment epithelium (RPE) in unison with changes to underlying Bruch’s membrane leading to deposition of sub-RPE drusen and basal laminar deposits, photo-oxidative lipofuscin and loss of retinal pigment [1]. While the pathogenesis of AMD remains ill-defined, it is recognised that age-related RPE dysfunction in combination with progressive inflammatory and oxidative damage are of central importance [2]. In the neovascular (wet) form of AMD (nvAMD) RPE-Bruch’s membrane damage is associated with choroidal neovascularisation (CNV) which is stimulated by pro-inflammatory cascades and macrophage infiltration in the outer retina [3].

RAGE is a member of the immunoglobulin super-family with a high affinity for several ligands including advanced glycation endproducts (AGEs), S100B, high-mobility group box-1 (HMGB-1), amyloid-β and Mac-1 [4]–[6]. As a component of the innate immune response, this receptor is expressed in many tissues and regulates a range of pathophysiological responses linked to pathways such as ERK1/2, MAP kinases, P38 and JAK/STAT, downstream activation of NFκB, induction of pro-inflammatory cytokines and generation of reactive oxygen species [7]. RAGE is associated with Alzheimer’s disease, cardiovascular disease and diabetic vasculopathy [8] and recent evidence also indicates that this receptor could play an important role in tumour angiogenesis [9], atherosclerosis [10] and some microvascular disorders [11].

In the normal retina, RAGE expression occurs predominantly in the Müller glia although levels may become elevated in diabetic conditions [12], [13]. RAGE is constitutively expressed on RPE and levels increase during age-related pathology, especially in cells adjacent to drusen [14]–[16]. Also in RPE in vitro, exposure to RAGE-ligands (AGEs or S100B) induces expression of angiogenic factors [17]. Many RAGE ligands occur in the retina as a normal consequence of ageing. For example, AGE-modified proteins accumulate at the RPE-Bruch’s membrane axis [18], [19] where they are likely to have an important pathogenic role in the development of AMD [18]. AGEs are also increased in RPE, drusen and Bruch’s membrane from ageing eyes and in patients with AMD and adduct formation has been linked with chronic inflammation at the outer retina [20].

S100B interactions with RAGE are crucial for microglial activation in inflammatory brain pathology although at low concentrations S100B functions as a neurotrophic factor independently of RAGE [21]. The importance of S100B in the retina is much less well understood although it occurs in glia and acts as a calcium regulator in association with photoreceptor guanylate cyclase [22]. Recent studies have also demonstrated that glial-linked S100B is increased in diabetic retina where it can regulate inflammatory signaling via RAGE [13]. Similar responses have been observed in retinal capillary endothelium [23]. In view of the connection between RAGE and inflammation, the current study has evaluated the hypothesis that this receptor and its interaction with S100B plays a role in CNV. Using experimental in vivo and in vitro approaches it is demonstrated that RAGE is associated with sight-threatening angiogenic pathology.

## Materials and Methods

### Mice and CNV Induction

Wild type (WT) C57BL/6J mice were purchased from Harlan Laboratories (UK) and maintained within the Biological Research Unit at Queen’s University Belfast. The RAGE knockout mouse (RAGE−/−) was generated as previously described [24], [25] on an SVEV129×C57BL/6 background (Taconic Inc, Germantown, NY) and backcrossed to C57BL/6 mice for 5 generations. These RAGE−/− mice were shown to possess the Crb1 gene Rd8 mutation via DNA sequencing. Because of the presence of the RD8, the retina of both WT and RAGE−/− mice were subjected to histological investigation and Spectral Domain Optical Coherence Topography (SD-OCT).

All experiments were performed in accordance with UK Home Office and University Ethics Committee guidelines (Animals [Scientific Procedures] Act, 1986). Specifically, Queen’s University Animal Ethics Committee approved this study. 12 weeks old, female WT and RAGE−/− mice were used for each experimental set (n = 12/group). To create CNV, animals were anaesthetized and rupture of Bruch’s membrane-choroid were achieved by laser photocoagulation (Haag Streit BM 900 Slit Lamp and Argon laser; Haag Streit, UK) using burns of 50 µm spot size (0.05 s duration, 250 mW) approximately 2 disc-diameters away from the optic disc. Following CNV induction, confocal scanning laser ophthalmoscopy (cSLO) (Heidelberg Engineering, UK) was used to obtain infrared fundus images of the retina and angiography was also conducted following intravenous injection of 10% sodium fluorescein.

Retinal cross-sections in RAGE−/− and WT mice were acquired using the Spectralis Heidelberg OCT system (Heidelberg Engineering, Heidelberg, Germany) at a 30° field of view. Mice were anesthetized and the pupils were dilated by 1% tropicamide and 2.5% Phenylephrine (Chauvin, Essex, UK). Corneas were kept lubricated during the imaging session. High-resolution scans were acquired at all four regions (dorsal, ventral, nasal and temporal regions).

### CNV Lesion Assessment

Mouse eyes were fixed in 4% paraformadehyde for overnight, dehydrated with graded ethanol, and embedded in paraffin. Complete sectioning of whole eyes were performed. 6 µm thickness of sections were collected at regular intervals and processed for hematoxylin and eosin (H&E) staining. 6 mice from 3 different litters for each age group were used for the purpose. Age matched C57BL/6J were served as control. For evaluation of CNV lesions, eyes were fixed in 4% PFA. Sections, retinal flatmounts or RPE/choroid/sclera flatmouts were assessed using isolectin B4, CD68. RAGE and S100B. Isolectin and CD68 positive microglia were divided into three basic morphological categories using established methods [26]. At 1 week post-CNV induction, WT and RAGE−/− mice were sacrificed and eyes were enucleated and fixed in 4% PFA. Posterior segment flat mounts with neural retinal intact or removed were stained with biotin conjugated isolectin B4 (Sigma, UK), CD68 (Abcam, Cambridge, UK) and S100B (Abcam, Cambridge, UK) and with the corresponding secondaries, including streptavidin Alexa Fluor 488 (Molecular Probes, Paisley, UK)) or Alexa Fluor 568 goat anti-rabbit IgG. DAPI or propidium iodide (Sigma, UK) was also added to locate the nuclear layers of the retina. Isolectin-localised lesions were visualisation by confocal laser microscopy (Eclipse TE2000-U confocal microscope, Nikon, UK). CNV lesion size was quantified by measuring isolectin-positive area (NIS Elements, Nikon, UK) of the RPE/Choroid/sclera flatmount.

In flatmounts with the retina intact, isolectin positive microglia within or adjacent to the CNV lesion were quantified. The total number of microglial cells counts were subdivided according to whether the cells displayed dendritic or amoeboid morphology, the latter indicating activation as previously described. The cells were assessed in at least four Z-series images per specimen using a Nikon Eclipse TE2000-U Confocal Microscope and divided into two basic morphological categories as previously described [27]. Flat-mounts from at least 6 separate animals/treatment were assessed and the total number of microglial counts were subdivided according to whether the cells displayed dendritic or amoeboid morphology, the latter indicating activation.

The eyes were enucleated at different time points post-lasering and fixed in 2% PFA/PBS for two hours before embedded in OCT for cryostat section. Sixteen µm thick cryosections were blocked with 10% BSA for 30 mins. Rabbit anti-mouse RAGE (1∶100, Millipore, UK). Rabbit anti-mouse S100B (1∶200, Abcam, UK), biotin conjugated CD68 (1∶200, BD biosciences, UK) were incubated with the sections for overnight at 4°C. After washing in PBS, these slides were incubated with goat anti-rabbit Alex Flour 594 (1∶200, Invitrogen, UK), goat anti-rabbit Alex Flour 488 (1∶200, Invitrogen, UK), streptavidin FITC (1∶200, Vector Lab, UK) or PI (1∶200, Invitrogen, UK) for one hour at room temperature. After washing in PBS, slides were covered with Vectashield mounting medium (Vector Lab, UK) and evaluated using confocal laser microscopy (Eclipse TE2000-U confocal microscope, Nikon, UK).

### Quantitative RT-PCR (qPCR)

As previously described [13], quantitative PCR was used to assess expression of VEGF, RAGE, S100B, CSF-1 (MCSF-1),MCP-1, IL-1β, TNFα, and IL-6 in retina from RAGE−/− and WT mice. Total RNA was extracted from freshly dissected retinas of RAGE−/− and WT mice with or without laser-induced CNV (n = 7 mice/group) using Tri-Reagent (Sigma, UK). The Qiagen Quantitect reverse transcription system (Qiagen, West Sussex, UK)) was used to synthesize cDNA, with 1 µg of RNA and random primers, according to the manufacturer’s instructions. qPCR was performed for quantitative analysis of mRNA expression as previously described [13]. Sequence-specific primers were designed using the program NCBI primer blast to amplify VEGF (Forward: 5′ AGGATGTCCTCACTCGGATG3′;. Reverse: 5′ TCTGGAAGTGAGGCCAATGTG3′), RAGE (Forward: 5′GCCCGGATTGGAGAGCCACTTG3′;. Reverse: 5′ GAGGTTGAGTGGCCAGGCGTGC3′), S100B (Forward: 5′ TGCATGCTGGTCCCTGAGAATACTG3′; Reverse 3′GGTCCCCTCCTGCAGGTATGCC3′), CSF-1 (MCSF-1) (Forward: 5′ ACTTGGAGCGGACAGCCCCTT3′; Reverse: TTGAAATACCGCGGGCCTCAGC3′), MCP-1 (Forward: 5′ TCACCTGCTGCTACTCATTCACCA3′; Reverse: 5′ AAAGGTGCTGAAGACCCTAGG GCA3′), IL-1β (Forward: 3′ GCCCATCCTCTGTGACTCAT3′; Reverse 5′ AGGCCACAGGTATTTTGTCC3′), TNFα (Forward: 5′ CGTCAGCCGATTTGCTATCT3′; Reverse: 5′ CGGACTCCGCAAAGTCTAAG3′), IL-6 (Forward: 5′ AGTTGCCTTCTTGGGACTGA3′; Reverse: 5′ TCCACGATTTCCCAGAGAAC3′) and 18s (Forward: 5′ GTAACCCGTTGAACCCCAT3′; Reverse: 5′ CCATCCAATCGGTAGTAGCG3′). Results were normalized to 18s.

### Macrophage Culture and Chemotaxis Assay

Mice were sacrificed in a CO2 chamber and hind limbs were collected. Bone marrow derived macrophages (BMDMs) from WT and RAGE−/− mice were cultured as described previously [28] with slight modification. Briefly, femoral bone marrow cells were cultured in 75mm^2^ culture flasks in DMEM supplement with 10% heat-inactivated FBS with 10% LADMAC supernatants, which served as a source of M-CSF. Medium were changed as required. Seven days later, macrophages were collected for experiments. Chemotaxis assays were performed using the Boyden Chamber system with modifications as described previously [28]. 5×10^5^ single cell suspension of BMDMs were seeded into 5.0 µm pore size of Transwell® (Corning B.V. Life Sciences, Amsterdam, Netherland) and cultured with different concentration of S100B (Millipore, UK) for 20 hrs. Migrated cells from inserts to the bottom chamber were counted. Five images were taken from the centre, left, right, superior and inferior fields of view of the chamber using an inverted microscopy. The average number of cells from the five images was taken to represent the number of migrated cells. All experiments were performed a minimum of three times.

#### Statistical analysis

Student t test is used for two groups comparison. For multiple groups comparison, a one way ANOVA were performed first, then a Newman-Keuls multiple comparison test were followed using Graph pad InStat 3.0 (GraphPad Software, San Diego, CA, USA). All data were considered significant at a level of p<0.05 and presented as mean +/−SEM.

## Results

### Retinal Degeneration is Not Present on WT or RAGE−/− Mice

**Figure 1:**
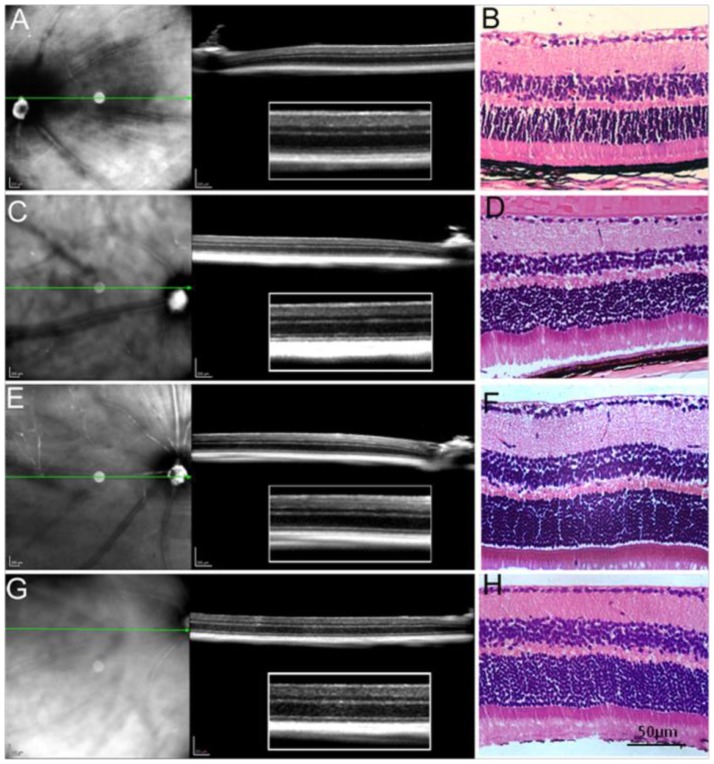
SD-OCT and histologial analysis of retina from one month RAGE −/− mice (A, B), 9-month old RAGE −/− mice (C, D), one month old C57BL/6J mice (E, F) and 9-month old C57BL/6J mice (G, H). Retinal sections shows well-defined retinal structures from both RAGE−/− and C57BL/6J mice. Scale bar on A, C, D and F is 200 µm. The scale bar on B,D,E and F is 50 µm.

Typically, mice homozygous for Crb1^rd8^ mutation exhibit large white retinal spots in the fundus images and a slow progressive retinal degeneration [29], [30]. However, not every strain which has the rd8 mutation develops a retinal degeneration phenotype. For example, Chang et al has demonstrated that only 23 strains out of 83 strains with the rd8 mutation demonstrated retinal degeneration [29]. We studied the histology and OCT of eyes of different ages of RAGE−/− mice (Fig. 1). The OCT retinal cross sections showed hyper-reflective or hyporeflective interfaces representing the normal layers of retinas. Eye sections showed normal architecture with well-defined nuclear layer (GCL, INL and ONL), synaptic layers, intact photoreceptors (inner and outer segments) and the RPE. We were unable to detect any patches of dysplasia, retinal folds or pseudorosettes which is morphology typical of retinal degeneration caused by the rd8 mutation [31].

### RAGE/ligand Axis Expression is Increased in CNV Development

**Figure 2:**
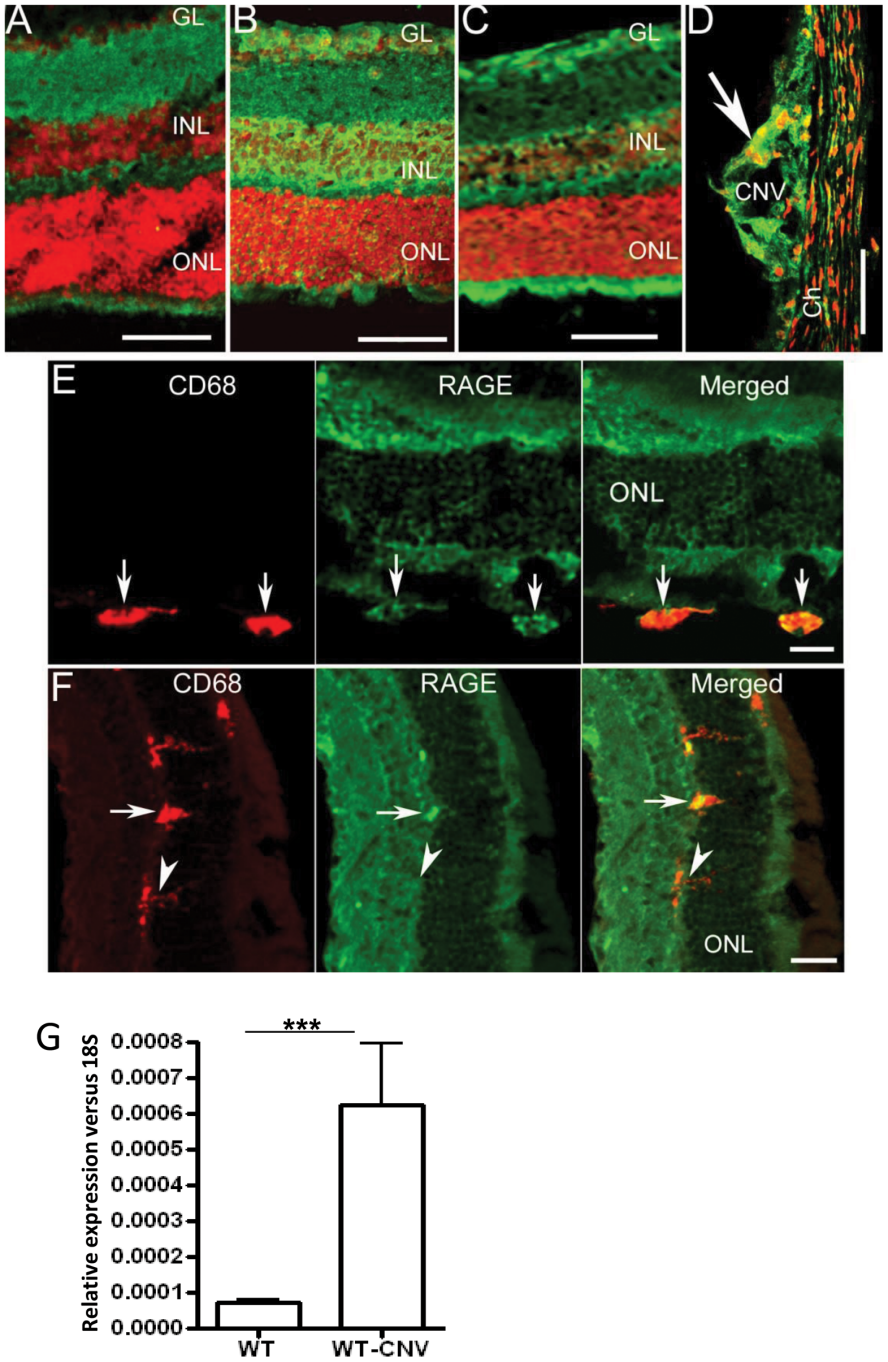
Rage expression in laser-treated eyes. Representative images demonstrating the RAGE expression at the eye sections from control (A), day 1(B) day 5 (C) and day 7 (D) post-lasering in WT mice. Scale bar = 50 µm. E) Retinal section from day 5 post-laser mouse eye showing macrophage (arrows) at the subretinal space express CD68 (red) and RAGE (green). Scale bar = 20 µm. F) Retinal section from day 5 post-laser mouse eye showing CD68+ microglia at the ONL can be RAGE positive (arrow) or RAGE (arrowhead). Scale bar = 20 µm. G) RAGE mRNA expression in the normal WT retina and laser-treated retina. RAGE mRNA level was significantly increased following laser treatment (n = 7 mice/group, P<0.001).

To determine the involvement of RAGE in CNV development, eye sections were stained with RAGE antibody at different time points post lasering in WT mice. Low levels of RAGE expression were detected in the neuronal retina in the inner plexiform layer, outer plexiform layer and in the inner segments of photoreceptor (Fig. 2A). One day after laser treatment, the expression of RAGE was markedly increased (Fig. 2B) and the expression levels remained at high levels through to day 7 (Fig. 2C). RAGE-immunoreactivity was also detected at the site of CNV (Fig. 2D). Dual staining of CD68 and RAGE showed that RAGE is also expressed by infiltrating CD68^+^ subretinal macrophages (Fig. 2E). In the inner retina, activated microglia also expressed CD68 (Fig. 2F). Some of these CD68^+^ microglia expressed RAGE (arrow, Fig. 2F) while other cells were negative for CD68 (arrowhead, Fig. 2F). Furthermore, RAGE mRNA levels were significantly increased in the retina following CNV induction (p<0.001) (Fig. 2G).

**Figure 3:**
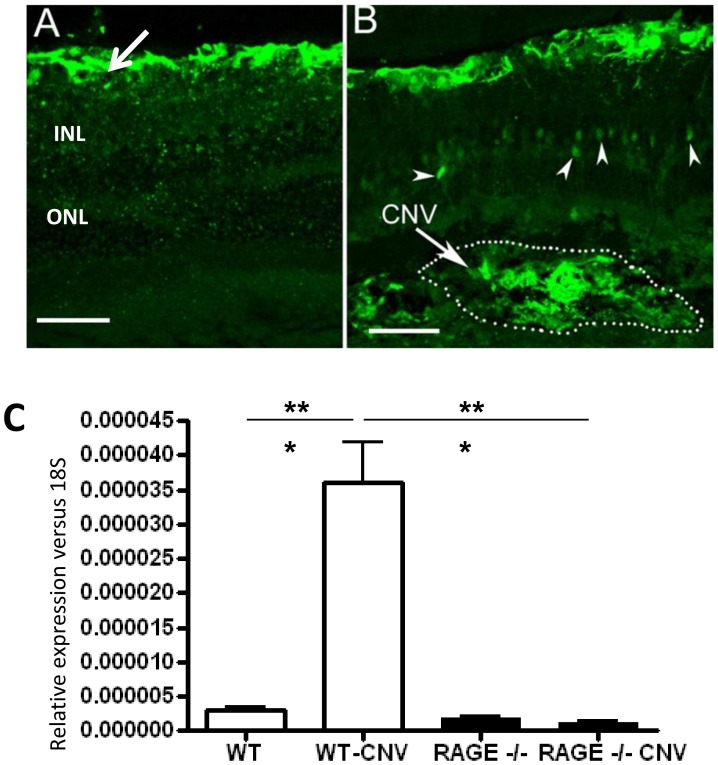
S100B is present in CNV lesions. A) S100B expression in a normal WT mouse retina. Strong immunoreactivity is present in the astrocytes (arrow). The position of the inner nuclear layer (INL) and outer nuclear layer (ONL) are indicted. Scale bar is 50 µm B) S100B expression in WT mouse retinal at day 7 post-laser treatment. S100B was detected in the outer plexiform layer (arrowheads). Strong S100B expression was detected at the site of CNV. Scale bar is 50 µm C) Real-time PCR demonstrates that S100B mRNA is significantly upregulated in retina with laser-induced CNV lesions (n = 7 mice/group ***P<0.001). This increase is not observed in RAGE−/− mice.

S100B is one of the main ligands of RAGE and under normal physiological conditions this protein is expressed by astrocytes in the retina [13] (Fig. 3A). However, following CNV induction in WT retina, the expression of S100B was increased and could be detected in the outer plexiform layer (arrowheads in Fig. 3B). Furthermore, strong S100B expression were detected at the site of CNV (Fig. 3B). S100B was significantly up-regulated in the lasered WT retina when compared to non-lasered control retina (P<0.001, Fig. 3C).

### Genetic Deletion of RAGE Reduces CNV Lesion Size

**Figure 4:**
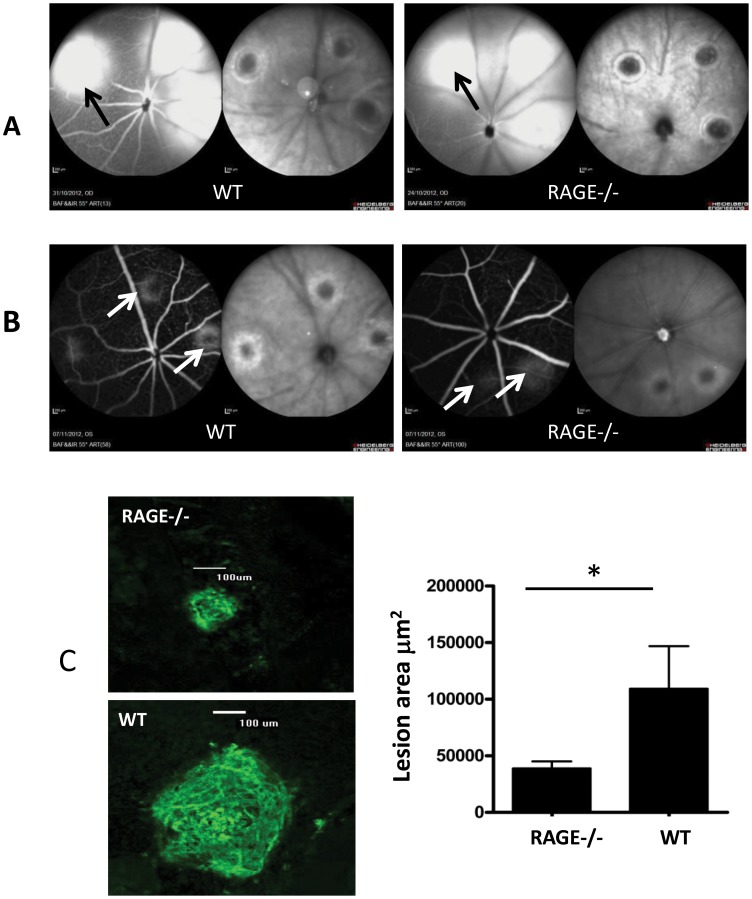
Laser-induced CNV lesions are attenuated in RAGE−/− mice. Representative images demonstrating laser-burned spots immediately (A) and 7 days after photocoagulation (B) in RAGE−/− mice and WT controls. A) As assessed by cSLO, the retina from WT and RAGE−/− mice shows laser burn sites immediately after photocoagulation. The left image in each pair is fluorescein angiography and the right image is infrared reflectance. There is no obvious difference between the two animal groups with comparable leakage at the lesion (black arrow). B) 7 days after photocoagulation the CNV lesions are apparent in angiograms and infrared reflectance fundus images from WT mice although these are smaller in the retina of RAGE−/− mice (white arrows). C) Comparison of CNV lesion size between the WT and RAGE−/− mice. Retinal flat mounts were evaluated for the presence and size of clearly demarcated isolectin positive CNV lesions one week post-laser injury. RAGE −/− mice exhibited significantly less CNV than age matched controls (n = 12 animals/group, *p<0.05) (Scale bar = 100 µm).

RAGE expression is increased in the retina following CNV induction. In particular, we have shown that there is increased RAGE expression in infiltrating macrophage and local resident macrophages (microglia). Therefore, we hypothesised that the RAGE pathway may be involved in the development of CNV. To test this, we studied the effect of RAGE deficiency on CNV lesions. CNV was produced in 12 week-old WT and RAGE−/− mice. Upon examination by fundus photography and fluorescein angiography there was no difference in the nature of the laser burn and the associated leakage at day 0 between C67BL/6 and RAGE−/− mice (Figure 4A). Seven days post lasering, angiography revealed a marked difference in the size of laser-induced CNV lesions in the retina of RAGE−/− mice when compared to WT controls (Figure 4B). Post-mortem evaluation using confocal microscopy of retinal flat-mounts, stained with isolectin B4, demonstrated a significant reduction in CNV lesion size in the RAGE−/− mice when compared to WT counterparts (P<0.05) (Figure 4C). The S100B expression did not show any alteration in RAGE−/− which had CNV induced (Fig. 3C).

### Reduced Retinal Immune Cell Activation and Cytokine Expression in RAGE−/− Mice

**Figure 5:**
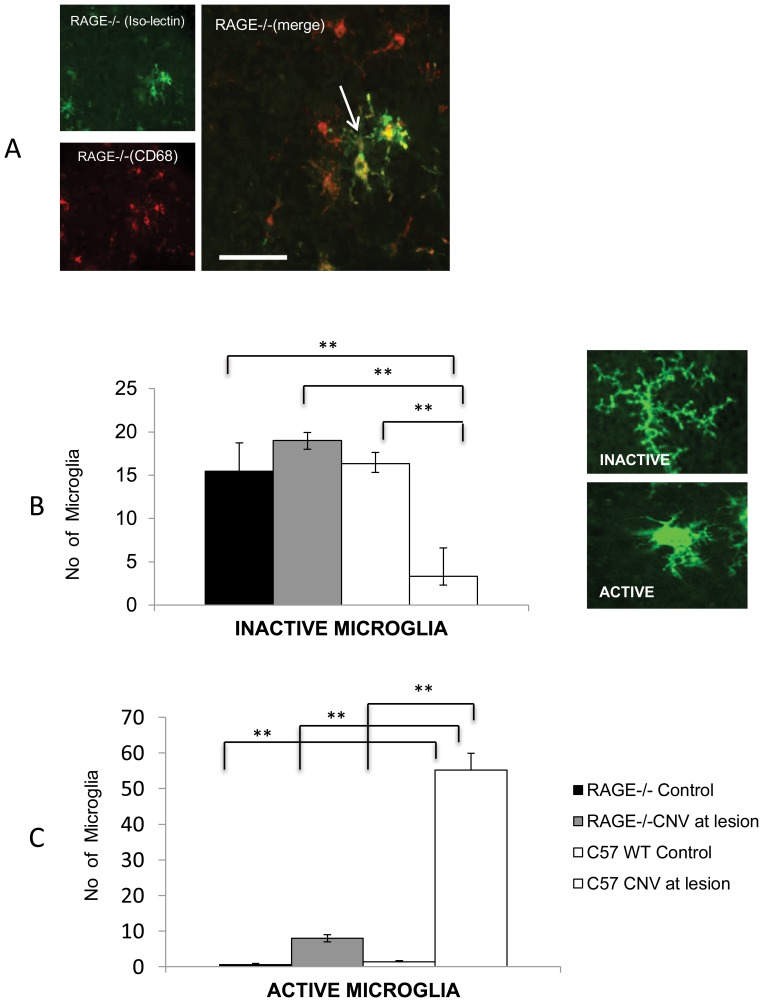
RAGE regulates infiltration of microglia/macrophages to CNV lesions. A) The macrophage marker CD68 and isolectin (which binds to microglia and macrophages) were used to assess immune cell infiltration into CNV lesions within the retina of WT and RAGE−/− mice. Representative images demonstrated that Isolectin-positvie cells sometimes co-locaslised with CD68 positive cells. Most isolectin-positive cells demonstrated a dendritic morphology (arrow). (Scale bar = 50 µm). B) Microglia were quantified both in CNV lesions from WT and RAGE−/− mice. Isolectin-positive cells with a typically dendritic (inactive) morphology occurred at high numbers in retinas of non-lasered mice but only in the WT did laser cause a reduction in this phenotype. (** P<0.01). Representative images are shown of typical dendritic and amoeboid microglial cell in the retina. C). Active microglia (with an amoeboid phenotype) were higher in lasered WT and RAGE−/− mice when compared to their non-lesioned controls. RAGE−/− mice had significantly fewer active microglia compared to WT mice (**P<0.01). n = 12 mice/group.

RAGE is expressed by macrophages [32](Fig. 1E and 1F), and infiltrating macrophages are known to play a critical role in CNV formation [33], [34]. To understand whether reduced CNV in RAGE−/− mice is related to macrophage infiltration/activation, we then examined CNV-related immune cell function. Isolectin and CD68 positive cells were present in the CNV lesions of both WT and RAGE−/− mice and both markers were often co-localised, although some cells were present that expressed CD68 alone (Figure 5A). Isolectin-positive microglia or infiltrating macrophages were quantified at the CNV lesion and also in remote (non-lasered) retina. Isolectin-positive cells with a dendritic phenotype were present in the non-lasered retina (remote from the CNV lesion) and there was no difference between WT and RAGE−/− animals (Figure 5B). However at the CNV lesions, WT animals displayed a significant shift towards amoeboid (activated) cells when compared to non-lasered regions ((P<0.01) (Figure 5C). RAGE−/− animals also had more amoeboid cells at CNV lesions but these were significantly less when compared to WT counterparts (P<0.01) (Figure 5C).

**Figure 6:**
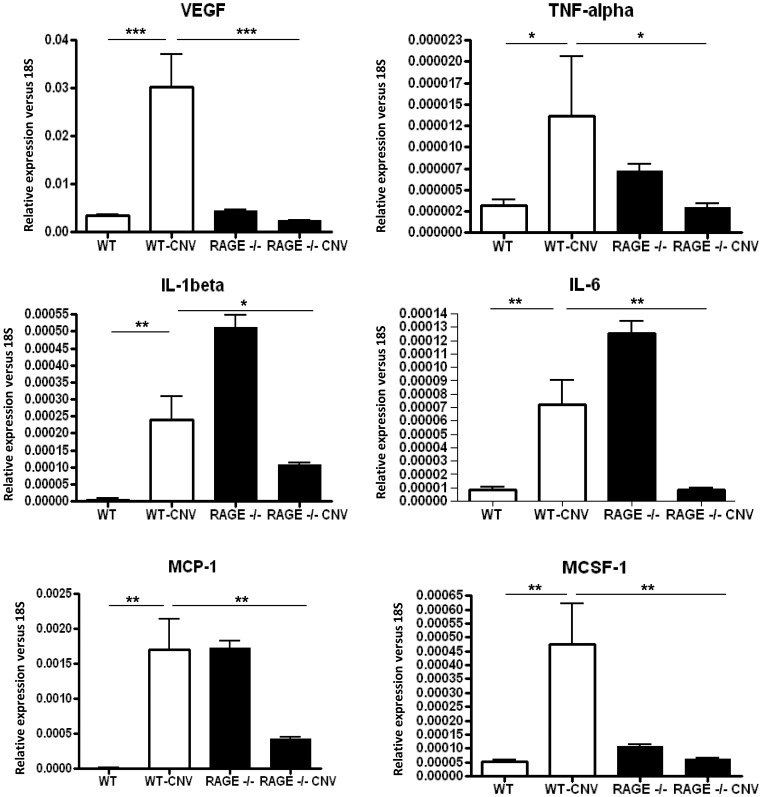
RAGE regulates growth factor and cytokine expression during CNV. CNV lesion induction in WT mice produces a profound growth factor and cytokine response with VEGF, TNF-α, IL-1β, IL-6, MCP-1 and MCSF-1 all showing a significant up-regulation in comparison to control (non-lasered) retina. The retina of RAGE−/− animals appears to show a constitutively higher level of some cytokines when compared to WT (IL-1β, IL-6 and MCP-1). Upon laser treatment, the retina from RAGE−/− mouse retina shows a significantly suppressed VEGF and cytokine response when compared to WT. (n = 7 mice/group, *P<0.05; **P<0.01; ***P<0.001).

In view of immune cell infiltration to the CNV lesions, retinal mRNA expression of pro-inflammatory cytokines and VEGF was investigated in the WT and RAGE−/− groups (Figure 5). VEGF was significantly increased in the WT retina where CNV was present (P<0.001) although this response was significantly suppressed in the RAGE−/− mice (Figure 6). CNV also induced mRNA expression in TNF-α, IL-1β, IL-6, MCP-1 and MCSF-1 when compared to normal, non-lasered retina (P<0.05–0.01)(Figure 6). While baseline levels of some cytokine transcripts were higher in RAGE−/− mice when compared to WT (IL-1β, IL-6 and MCP-1), there were consistently blunted CNV-induced cytokine responses in the RAGE−/− mice and in many cases expression was reduced (Figure 6).

### S100b Induces Macrophage Chemotaxis

**Figure 7:**
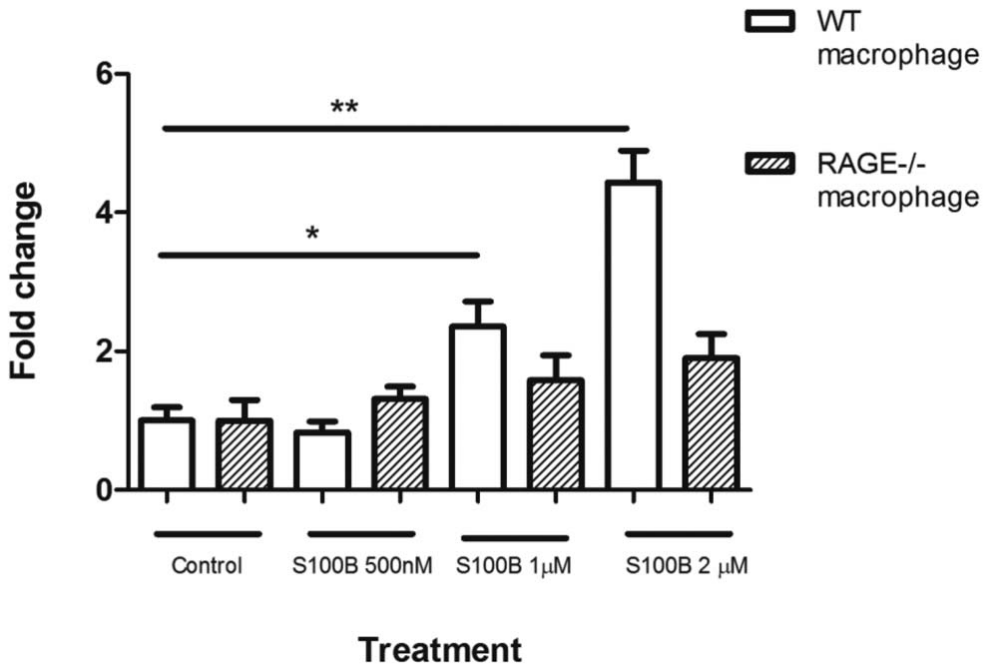
Chemotaxis of bone marrow-derived macrophages (BMDMs). Chemotaxis assay was carried out using the Boyden chamber system. Migration of BMDMs of WT and RAGE−/− mice in response to S100B at different concentrations. * P<0.05; **P<0.01.

Our results show that RAGE and S100B were increased in laser-induced CNV and deletion of RAGE reduced lesion size which was accompanied by decreased immune cell activation/infiltration. This suggests that the RAGE-S100B pathway may play an important role in macrophage infiltration following CNV induction. To further test this observation, we conducted a chemotaxis assay using BMDMs isolated from from WT and RAGE−/− mice. Cells from both donor mice had similar levels of random migratory activity under normal culture conditions (Fig. 7). However, in response to S100B, a dose-dependent chemotactic migration was observed in BMDMs from WT mice (p<0.05–0.001) but this response was not apparent in cells isolated from RAGE−/− mice (Fig. 7).

## Discussion

RAGE plays an important role in inflammatory responses in diseases such as Alzheimer’s, diabetic complications and acute lung injury [7]. Its sustained activation can change an acute inflammatory response into prolonged inflammation that culminates in tissue damage [8] and, in some cases, immune cell-mediated angiogenesis [35], [36]. The current study has demonstrated, for the first time, that RAGE could play an important contributory role in immune cell activation within CNV lesions.

The reduction of CNV lesion size in RAGE−/− mice is a key finding of this study. While such laser-induced lesions do not involve age-related pathology, this model is widely used to reproduce key aspects of nvAMD [37]. S100B attracts macrophages to sites of injury and interestingly, RAGE−/− mice have this pro-inflammatory protein present at CNV lesions although they demonstrate reduced immune cell infiltration and no enhancement of pro-inflammatory cytokine expression. This is probably due to suppressed S100B-RAGE activation and coincides with a recent *in vitro* study in microglia demonstrating a critical role for RAGE in S100B-mediated migration and chemokine release [38]. S100B is a neurotrophic factor that regulates cytosolic Ca2+ and cytoskeletal integrity in astrocytes, oligodendrocytes, neural progenitors, Langerhans cells and dendritic cells [21]. When it is secreted extracellularly S100B is a potent pro-inflammatory factor that can activate macrophages and evoke tissue damage [39]. In the retina, S100B is constitutively expressed by astrocytes and Müller glia [22] although it is upregulated in conditions such as diabetes [13] where it can provoke inflammatory cytokine expression via RAGE-activation of p44/42, p38, JNK and/or p90RSK [40].

The current study shows that the retinal expression of some cytokines were at significantly higher baseline levels in RAGE−/− mice when compared to WT controls, an observation that has been previously reported [24]. However, upon CNV induction, there is no further increase in the expression of these cytokines and often there is a significant decrease. The underlying reason for this is unclear although the laser-induced immune response in the RAGE−/− may be shifted from a pro-inflammatory to wound-healing/anti-inflammatory response with accompanying changes in ratios of M1 to M2 macrophage phenotypes in the lesion area. In addition, it may be related to the regulatory role of RAGE in pro-inflammatory networks and dependency of NF-κBp65. Absence of RAGE may initiate different composition of NF-κB complexes in the absence of RAGE and/or the interaction of NF-κB with different (e.g. compensatory) cofactors could account for this phenomenon.

As a pattern-recognition receptor, RAGE binds many ligands and several of these are relevant to the retina. For example, AGEs occur in the ageing retina and these adducts are associated with AMD [14]. While not a focus for the current study, AGEs could also play an important regulatory role in RAGE activation, especially since they accumulate at the RPE-Bruch’s membrane axis [41], [42]. In the future, it would be important to determine how RAGE could regulate pathology in ageing murine models which show lesions characteristic of early-stage AMD. Related to this is evidence that toll-like receptors (TLRs) play a role in inflammation-mediated pathology [43] and may be involved in retinopathy [44], [45]. The interaction between TLRs and RAGE is potentially important and could contribute to innate immune responses in the retina, especially since they share common ligand interactions with S100 and HMGB1 [46].

Inflammation at the outer retina plays a major role in the development of neovascular pathology in AMD [47]. Indeed, activation of resident microglia and infiltration of immune cells into the subretinal space are important aspects of the laser-induced CNV response in mice [48], [49] and it has been suggested that these cells may be a driving force in the pathogenesis of AMD and not simply a secondary consequence of primary RPE or photoreceptor disease [50]. More recently, it has been suggested that a critical population of infiltrating pro-inflammatory cells are M1 macrophages and contribute to AMD-like degenerative pathology in a murine model [51]. RAGE plays a role regulation of monocyte recruitment across the vasculature and into the inflamed brain [52] and this receptor has been linked to migratory influx of immune cells into tissues in association with up-regulation adhesion molecules interactions [52], [53]. In the current study we show that S100B is a key stimulatory molecule that induces migration of BMDMs and that this response was absent when RAGE was deleted. This “ex vivo” assessment corresponds closely to the observed responses of infiltration of CD68+ cells in the retina and activation of microglia in the retina post-CNV. The role of RAGE on macrophage polarity is not well established, although a recent study has suggested that S100B was secreted from adipocytes and correlated with up-regulation of M1 markers in RAW macrophages, a response prevented by RAGE neutralization [54]. It would be worthwhile to dissect the relative contribution of RAGE to immune cell infiltration in the context of CNV, AMD and other retinal diseases that involve inflammation. and these studies are currently ongoing in our laboratory.

Despite our mice showing no outer retinal degeneration, we were concerned that the presence of the rd8 mutation (as a Crb1 deletion) could interfere with development of CNV. While phenotype with the rd8 mutation is variable [29], CCL2(−/−)/Cx3cr1(−/−)/Crb1 [55] and C57BL/6 CD11c-eYFP mice [56] show profound photoreceptor degeneration. This is accompanied by enhanced macrophage activity in the outer retina indicating that inflammation occurs in the presence of the rd8 mutation. The development of CNV in animals requires infiltration of macrophages [57], [58] and it could, perhaps, have been anticipated that rd8 mutation would exacerbate laser-induced CNV in the RAGE−/− mice. In fact our data shows the opposite response. We feel that the absence of photoreceptor degeneration in our mice suggests that the mutation is minimally active and thus has not played a major role in the reduced size of CNV in RAGE−/− mice.

In summary, this investigation has demonstrated that RAGE activation by S100B contributes to CNV by regulating angiogenic activity, infiltration of immune cells to the lesion site and up-regulation of pro-inflammatory cytokines. Our data builds on previous reports that RAGE and some of its ligands are present in the outer retina of aged patients [59] and provides compelling evidence that use of agents that can attenuate RAGE signaling could have utility for preventing the most visually disabling manifestation of AMD.
